# Omega-3 Polyunsaturated Fatty Acid Supplementation for Reducing Muscle Soreness after Eccentric Exercise: A Systematic Review and Meta-Analysis of Randomized Controlled Trials

**DOI:** 10.1155/2020/8062017

**Published:** 2020-04-20

**Authors:** Zheng-tao Lv, Jin-ming Zhang, Wen-tao Zhu

**Affiliations:** Department of Orthopedics, Tongji Hospital, Tongji Medical College, Huazhong University of Science and Technology, Wuhan 430030, China

## Abstract

**Purpose:**

This systematic review and meta-analysis was performed to determine the effectiveness of Omega-3 polyunsaturated fatty acid (*n*‐3 PUFA) supplement on muscle soreness after eccentric exercise.

**Methods:**

PubMed, EMBASE, CENTRAL, and ISI Web of Science were searched to identify randomized controlled trials (RCTs) that assessed the efficacy of *n*‐3 PUFA on muscle soreness after eccentric exercise. Mean difference (MD) and the associated 95% confidence interval (95% CI) were calculated by RevMan 5.3 to indicate delayed onset muscle soreness (DOMS) that measured two days after eccentric trainings. Subgroup analyses according to duration and daily dosage of *n*‐3 PUFA supplements before eccentric exercises were performed to determine whether these factors will influence the overall effect size. The Grading of Recommendations, Assessment, Development, and Evaluation (GRADE) approach was used to evaluate the certainty of evidence. The protocol of this systematic review and meta-analysis was registered at PROSPERO (CRD42018085869).

**Results:**

12 RCTs containing 145 subjects and 156 controls were included in this study. Meta-analysis revealed a significantly decreased DOMS (MD -0.93; 95% CI -1.44, -0.42; *P* = 0.0004) in *n*‐3 PUFA supplement groups, while no significant differences in isometric muscle strength and range of motion (ROM) were detected. However, the pooled effect size for DOMS was lower than the minimal clinically important difference (MCID) of 1.4 on the 10-unit VAS, suggesting that the effect size of less muscle soreness with *n*‐3 PUFA supplements did not appear to be clinically relevant.

**Conclusion:**

There is low-quality evidence that *n*‐3 PUFA supplementation does not result in a clinically important reduction of muscle soreness after eccentric exercise. Isometric muscle soreness and range of motion were not improved by *n*‐3 PUFA supplementation either (low-quality evidence). To further elucidate the overall role of *n*‐3 PUFA on muscle damage in this area, large-scale RCTs are still needed.

## 1. Introduction

Omega-3 polyunsaturated fatty acids (*n*‐3 PUFAs) are essential fatty acids rich in fish oils. The predominant components of *n*‐3 PUFA including eicosapentaenoic acid (EPA), docosahexaenoic acid (DHA), and alpha-linolenic acid (ALA), which can reduce the production and release of arachidonic acid and proinflammatory prostaglandins (PGs), are believed to have potent anti-inflammatory effects. Nutritional intervention with *n*‐3 PUFA is widely believed to have the potential to alter the trajectory of a wide range of diseases including autoimmune diseases such as rheumatoid arthritis [1], inflammatory bowel diseases [2–5], and lupus nephritis [6], as well as cancer [7–10], cardiovascular diseases [11–14], metabolic disorders [15], and even neuropsychiatric decrements [16–18]. As for healthy individuals, current literatures focus on its implicit roles in metabolism [19] as well as skeletal muscle functions [20, 21], among which a number of researches have also revealed its temporal effects in skeletal muscle, and even implied that it may play a role in delayed onset muscle soreness (DOMS) after eccentric exercise.

DOMS is recognized as a kind of common muscle discomfort occurring after unfamiliar exercises especially when a person is repeatedly exposed to high eccentric muscle contractions or unaccustomed exercise [22]. It increases within the first 24 h postexercise, and rises to peak between 24 to 48 hours [23–26] or 48 to 72 hours [23, 27] according to different studies, then subsides and eventually disappears in 5 to 7 days [23, 28]. Similar to acute muscle soreness, it presents with muscle stiffness, aching pain, and/or muscular tenderness [28–30]. Though the exact physiological mechanism of DOMS remains equivocal, researches have revealed its relations with damage of sarcomeres, ensuing swelling of damaged muscle fibers, and subsequent initiation of an inflammatory response [31–34]. So far, physical therapies, including postexercise massage [35, 36], cryotherapy [37], low-level phototherapy [38], and vibration therapy [39, 40], as well as the application of pharmacologic nonsteroidal anti-inflammatory drugs (NSAIDs) [41–46], are the predominant means employed to alleviate DOMS. Actually, since DOMS is widely accepted as a common self-treated condition, current studies have been turning to dietary interference, such as caffeine [47], ginger [48, 49], and taurine [50, 51], and some studies also revealed *n*‐3 PUFA as a potential therapeutic agent to blunt DOMS. Despite the fact that majority of these studies are randomized controlled trials (RCTs), their sample sizes are relatively small, which though seems to be a common fault of trials in sports medicine, could not afford adequate statistical power. Therefore, to further elucidate the gainful benefit of *n*‐3 PUFA in this field, we conducted a systematic review and meta-analysis based on the variable outcomes from current evidences.

## 2. Methods

This systematic review and meta-analysis was performed and reported following the Preferred Reporting Items for Systematic reviews and Meta-Analyses (PRISMA) guideline [52]. The protocol for this study was registered with PROSPERO (CRD42018085869).

### 2.1. Literature Search Strategy

A thorough literature search was conducted by searching PubMed, EMBASE, CENTRAL, and ISI Web of Science to identify potentially eligible articles about *n*‐3 PUFA supplementation and muscle soreness after eccentric exercises. Four electronic databases were searched from their inception date to the latest issue (June, 2018) without language restriction. A combination of medical subject headings (MeSHs) and free terms were used based on the specifications of each database. An example of literature search strategy for PubMed was as follows: (“Fatty Acids, Omega-3”[MeSH] or omega-3 or *n*‐3 polyunsaturated fatty acid or *n*‐3 PUFA or “Eicosapentaenoic Acid”[MeSH] or Eicosapentaenoic Acid or EPA or “alpha-Linolenic Acid”[MeSH] or alpha-Linolenic Acid or Linolenic acid or “Docosahexaenoic Acids”[MeSH] or Docosahexaenoic Acids or DHA or “Fish Oils”[MeSH] or fish oil or fish oils) and (randomized controlled trial or random or randomly or placebo or controlled trial) and (eccentric contraction or eccentric training or eccentric exercise). The bibliographies of related systematic reviews were also searched. In addition, the reference section for each included study was also manually searched for inclusion of further eligible studies.

### 2.2. Inclusion and Exclusion Criteria

#### 2.2.1. Types of Participants

Subjects enrolled in our systematic review were required to be adults that undertook eccentric exercise to induce muscle soreness. No restrictions on race, gender, and specific type of eccentric exercise were imposed. Volunteers were excluded if they consumed any kind of *n*‐3 PUFA supplementation within the last 6 months. Participants were also excluded if they had engaged in specific upper limb or lower limb eccentric training or other kinds of resistance training in the previous 6 months.

#### 2.2.2. Types of Intervention

Participants were required to take *n*‐3 PUFA supplements before and/or after a bout of eccentric exercise. The main components of supplements consumed should be *n*‐3 PUFA (i.e., EPA, DHA, or linolenic acid), and a combination of EPA, DHA, and linolenic acid was deemed eligible for inclusion in our study.

#### 2.2.3. Types of Control

Participants in control groups were required to take placebo capsules that did not contain*n*‐3PUFA. The main component of placebo capsules could be sunflower oil, corn oil, rice powder, and so forth. Both *n*‐3 PUFA capsules and placebo capsules ought to be identical in appearance.

#### 2.2.4. Types of Outcome

The primary outcome of our study was DOMS after eccentric exercises, measured using the VAS; secondary outcomes included joint range of motion (ROM), isometric muscle strength, MVC, upper arm/thigh circumferences, skin temperature, jump performance, and M-wave latency as well as blood markers such as serum CK, CRP, IL-6, TNF-alpha, IL-1ra, cortisol, iron, and LDH measured after eccentric trainings. Considering DOMS and inflammatory reaction in response to muscle damage training peak within 1 to 2 days or 2 to 3 days postexercise, according to different studies, we combined the data measured two days after the exercises.

#### 2.2.5. Types of Studies

Only RCTs that assessed the efficacy of *n*‐3 PUFA on muscle soreness after eccentric exercises were included. Non-RCTs such as case reports, case series, book chapters, and editorials were excluded.

### 2.3. Risk of Bias Assessment

In order to assess the risk of bias among our included studies, the Cochrane Collaboration's tool is utilized, which is based on seven items: random sequence generation, allocation concealment, blinding of participants and personnel, blinding of outcome assessment, incomplete outcome data, selective reporting, and other sources of bias. Two reviewers (ZL and JZ) judged the risk of bias among studies independently, and the results were compared afterwards. In case of disagreements regarding the risk of bias judgment, discussion was conducted until a consensus was reached. Only data from studies with low or unclear risk of bias can be included in the analysis.

### 2.4. Data Extraction

Two investigators (ZL and JZ) screened each article independently and were blinded to the findings of the other reviewer. Following the prespecified inclusion criteria, two reviewers performed a rigorous screening to identify eligible articles. Data were collected from these selected articles using a predetermined standardized data collection sheet, which included first author, year of the publication, country, sample size, demographic characteristics of participants in different groups, details of *n*‐3 PUFA supplementation and control, detailed protocol of eccentric exercise, and main outcomes.

Discrepancies between two reviewers were resolved through discussion until a general consensus could be reached. The third review author (WZ) was sought for opinions if a consensus could not be reached.

### 2.5. Data Synthesis

For DOMS and other continuous variables measured two days after eccentric trainings, mean difference (MD) and the associated 95% confidence interval (95% CI) were calculated using the Rev Man 5.3 (Copenhagen: The Nordic Cochrane Centre, The Cochrane Collaboration, 2014). Standard mean difference (SMD) and the associated 95% CI were calculated for continuous variables using the same methodology if the outcomes were measured using different ways. The chi-squared test and the Higgins *I*^2^ test were used to assess the heterogeneity among studies (*P* > 0.1 and *I*^2^ < 50% indicate acceptable heterogeneity) prior to the combination of data from an individual study [53]. Regardless of the between-study heterogeneity detected, the random-effect model was selected for statistical analysis because of the variety of types of eccentric exercises and dosage of *n*‐3 PUFA supplements among the included studies; the random-effect model is preferable in the presence or anticipation of interstudy variances [54].

Power analysis of our meta-analysis was performed using the Power and Sample Size Calculation (PS) program (https://biostat.mc.vanderbilt.edu/wiki/Main/PowerSampleSize) to evaluate whether our current study could offer adequate power to detect the beneficial effect of *n*‐3 PUFA on DOMS at a level of significance of 0.05. Subgroup analyses according to duration and daily dosage of *n*‐3 PUFA supplements before eccentric exercises were performed to determine whether these factors will influence the overall effect size. The leave-one-out sensitivity analysis was conducted by removing each included study in turn and reevaluating the resulting effect on pooled results to test the robustness of the summary estimate. Begg's rank correlation test and Egger's linear regression test as well as funnel plots using Stata version 12.0 (StataCorp LP, USA) were used to assess the publication bias [55].

### 2.6. GRADE Approach

The Grading of Recommendations, Assessment, Development, and Evaluation (GRADE) approach was used to evaluate the certainty of evidence for each outcome measurement [56]. In this approach, evidence from RCTs was initially regarded as “high quality” but could be downgraded to “moderate quality,” “low quality,” or “very low quality” depending on the presence and seriousness of five categories of limitations: limitations in the study design and implementation (risk of bias), indirectness of evidence, high and unexplained heterogeneity of results, imprecision of results, and high probability of publication bias. Two independent reviewers (ZL and JZ) finished the evaluation independently. Any discrepancy was resolved through discussion until a common consensus was reached. Otherwise, the third reviewer (WZ) was consulted for help. A table for the summary of findings was generated to explain the final results.

## 3. Results

### 3.1. Literature Search

**Figure 1:**
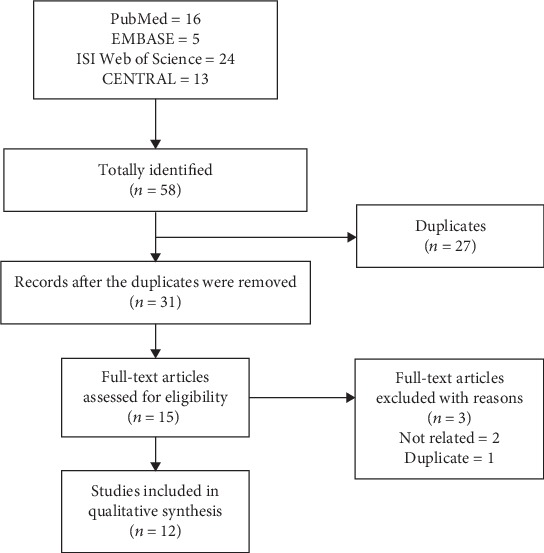
Flow diagram of literature search and screen.

An initial search yielded 58 potential citations, among which 16 were from PubMed, 5 were from EMBASE, 24 were from ISI Web of Science, and 13 were from CENTRAL. 27 citations were deleted because they were duplicates. After screening the titles and abstracts of the remaining 31 citations, 15 studies were selected and retrieved for full-text assessment based on the predetermined inclusion criteria, among which 3 were excluded with reasons, 2 were unrelated, and 1 contained duplicate data. Finally, a total of 12 RCTs [57–68] were considered eligible for inclusion and went into qualitative and quantitative syntheses. The literature screening process is presented in Figure 1.

### 3.2. Main Characteristics of Included RCTs

145 experimental subjects as well as 156 controls, covering Caucasian and Asian ethnic groups, regardless of genders, were included in this study. Of the 12 included trials, 5 were from USA [57, 58, 62, 64, 66], 3 were from UK [59–61], 2 were from Japan [65, 68], 1 was from Germany [67], and 1 was from Iran [63]. All trial results were published between 2002 and 2017, with individual sample sizes ranging from 14 to 41. Participants included were mostly young adults with ages ranging from 18.2 to 33.5 years old. Detailed information for *n*‐3 PUFA supplement and protocols of eccentric exercises, as well as main outcomes measured in each study are summarized in Table 1.

### 3.3. Risk of Bias

**Figure 2:**
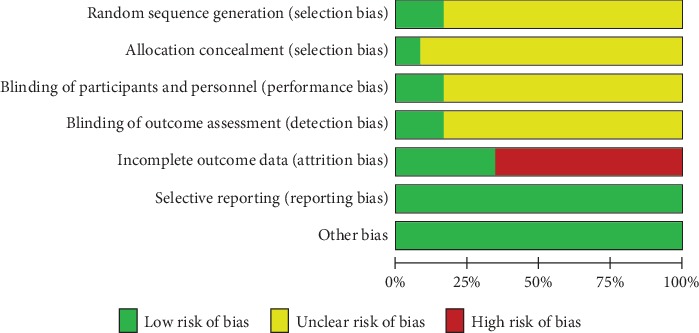
Risk of bias graph: review authors' judgments about each risk of bias item presented as percentages across all included studies.

**Figure 3:**
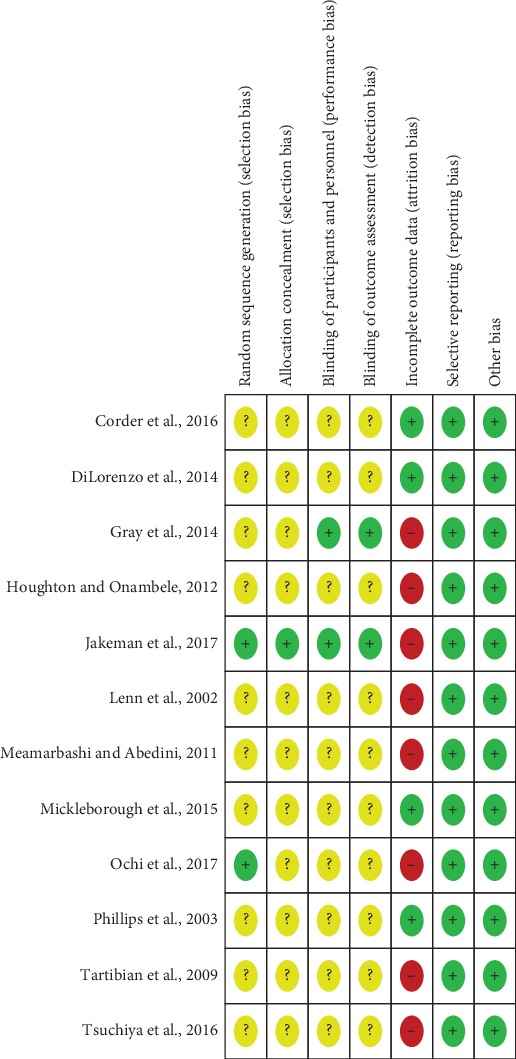
Risk of bias summary: review authors' judgments about each risk of bias item for each included study.

Cochrane Collaboration's tool was utilized for the assessment of risk of bias among included RCTs. All the studies selected in our study reported the suggested randomization, while only two studies reported the generation of random sequences. Details about allocation concealment were adequately provided by Jakeman et al. [61], whereas the other studies failed to report the procedure of allocation concealment. Although all the studies used placebo capsules that were identical with the *n*‐3 PUFA capsules in appearance, only two studies [59, 61] provided sufficient information about the blinding of participants and personnel and the blinding of outcome assessment. Only four studies [57, 58, 64, 66] reported adverse events that happened during the follow-up period, so the remaining eight studies were judged to have high risk of bias in terms of incomplete outcome data. Overall, four studies [57, 58, 64, 66] were judged to have moderate risk of bias, while the other eight studies were judged to have high risk of bias. The detailed information about reviewers' judgments about each risk of bias item is presented in Figures 2 and 3 .

### 3.4. Meta-Analysis Results of DOMS, Isometric Muscle Strength, and ROM

#### 3.4.1. Meta-Analysis Findings

**Figure 4:**
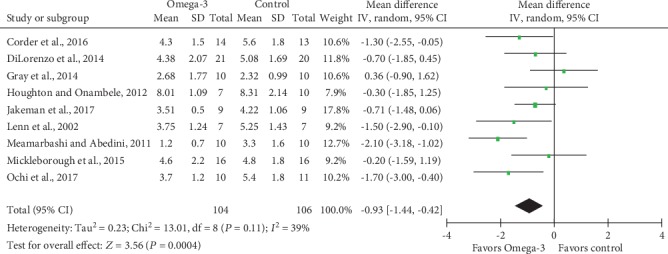
Forest plot of delayed onset muscle soreness: measured 2 days after eccentric exercise.

Meta-analysis of 9 studies that measured DOMS 2 days after eccentric exercises revealed significantly decreased VAS scores in *n*‐3 PUFA supplement groups compared to placebo controls (MD -0.93; 95% CI -1.44, -0.42; *P* = 0.0004; Figure 4); the random-effect model was selected as previously described. Based on the result of power analysis, our meta-analysis had a power of 0.942 to detect the gainful benefit of *n*‐3 PUFA on DOMS after eccentric exercise. Test for heterogeneity showed medium heterogeneity across included studies (Tau^2^ = 0.23; Chi^2^ = 13.01; df = 8; *P* = 0.11; *I*^2^ = 39%). In our current study, we used 1.4 as the minimal clinically important difference (MCID) for pain reduction based on the 10-unit VAS [69]. It should be noticed that the effect size of less muscle soreness with *n*‐3 PUFA was lower than the MCID, suggesting that the statistically significant difference in pain reduction was very unlikely to represent meaningful or important differences in clinical practice.

**Figure 5:**
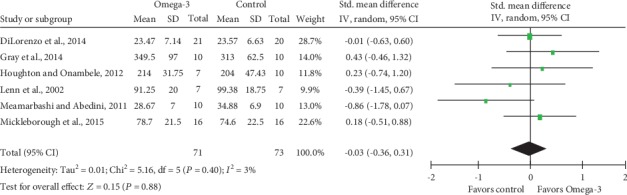
Forest plot of isometric muscle strength: measured 2 days after eccentric exercise.

**Figure 6:**
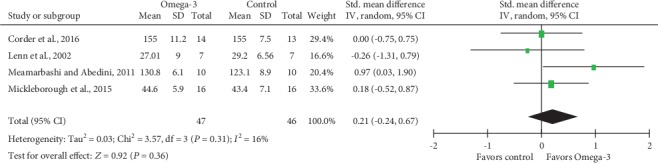
Forest plot of range of motion: measured 2 days after eccentric exercise.

Meta-analysis of isometric muscle strength (Figure 5) and ROM (Figure 6) demonstrated no statistically significant differences, with SMD and associated 95% CI -0.03 (95% CI -0.36, 0.31; *P* = 0.88) and 0.21 (95% CI -0.24, 0.67; *P* = 0.36), respectively. Besides, low heterogeneity was detected for analyses of both isometric muscle strength (Tau^2^ = 0.01; Chi^2^ = 5.16; df = 5; *P* = 0.40; *I*^2^ = 3%) and ROM (Tau^2^ = 0.03; Chi^2^ = 3.57; df = 3; *P* = 0.31; *I*^2^ = 16%).

#### 3.4.2. Subgroup Analysis

**Figure 7:**
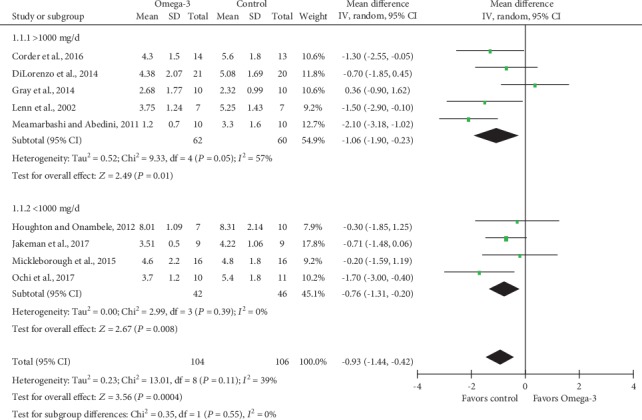
Forest plot of delayed onset muscle soreness: subgroup analysis by dose of Omega-3 supplementation.

**Figure 8:**
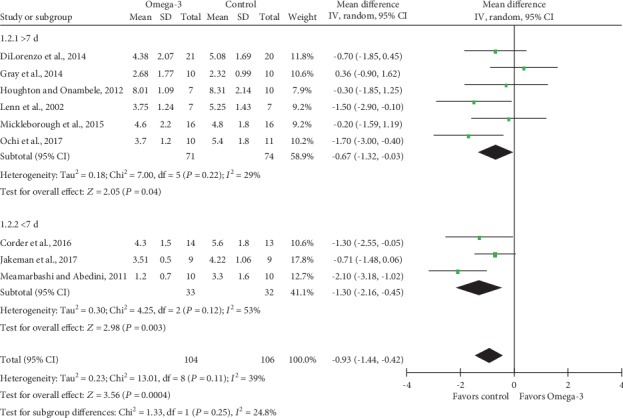
Forest plot of delayed onset muscle soreness: subgroup analysis by duration of Omega-3 supplementation before eccentric exercise.

Subgroup analysis of DOM was conducted based on *n*‐3 PUFA supplement dose stratification, and results indicated significantly decreased VAS scores in both >1000 mg/d and <1000 mg/d *n*‐3 PUFA intake groups, with MD and 95% CI -1.06 (95% CI -1.90, -0.23; *P* = 0.01) and -0.76 (95% CI -1.31, -0.20; *P* = 0.08), respectively (Figure 7). Heterogeneity test for subgroups indicated that there was no statistically significant heterogeneity (Chi^2^ = 0.35; df = 1; *P* = 0.55; *I*^2^ = 0%). We also performed subgroup analysis based on *n*‐3 PUFA supplement durations before eccentric exercises (Figure 8). MD and corresponding 95% CI for >7 d and <7 d subgroups were -1.06 (95% CI -1.90, -0.23; *P* = 0.01) and -1.30 (95% CI -2.16, -0.45; *P* = 0.003), respectively, which suggested significantly lower VAS scores in *n*‐3 PUFA groups regardless of supplement durations prior to eccentric exercises. Heterogeneity test revealed low heterogeneity between subgroups (Chi^2^ = 1.33; df = 1; *P* = 0.25; *I*^2^ = 24.8%).

#### 3.4.3. Publication Bias and Sensitivity Analysis

**Figure 9:**
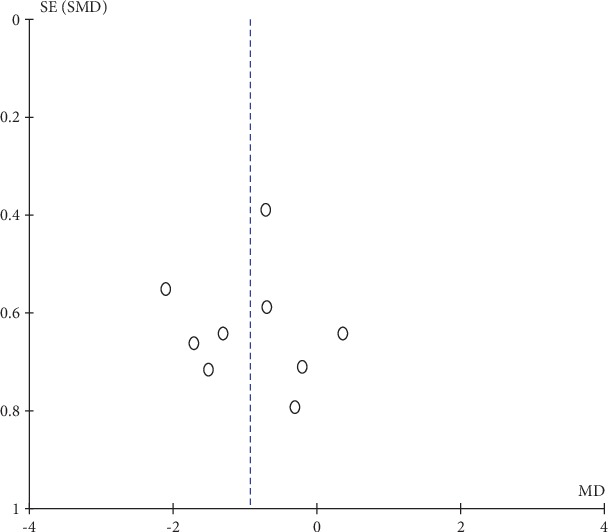
Funnel plot of delayed onset muscle soreness: measured 2 days after eccentric exercise.

**Figure 10:**
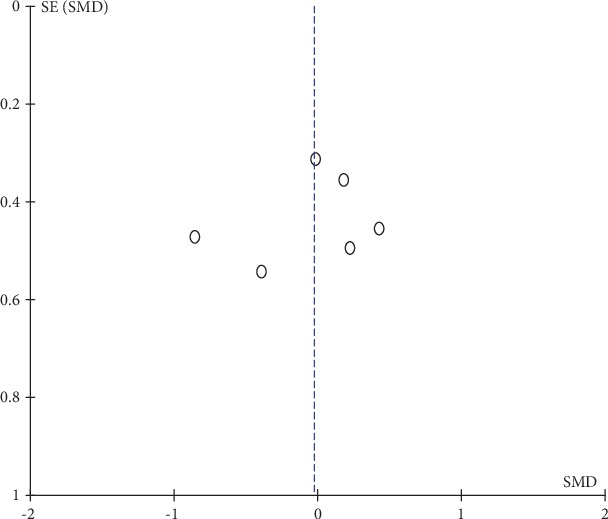
Funnel plot of isometric muscle strength: measured 2 days after eccentric exercise.

Funnel plots for DOMS and isometric muscle strength were visually symmetrical (Figures 9 and 10 ), which indicated acceptable publication bias; Begg's test (DOMS: *z* = 0.31, *P* = 0.754; isometric muscle strength: *z* = 0.38, *P* = 0.707) and Egger's test (DOMS: *t* = 0.10, *P* = 0.926; isometric muscle strength: *t* = −0.60, *P* = 0.583) also confirmed no statistically significant publication bias. A funnel plot was not generated for ROM because only 4 studies reported ROM as an outcome measurement. Sensitivity analysis confirmed the robustness of results of the meta-analysis because the removal of any individual study did not change the overall estimate (detailed data not shown).

### 3.5. Other Outcomes

#### 3.5.1. Blood Markers

Studies mainly focused on muscle damage markers like CK, lactate dehydrogenase (LDH), myoglobin, and iron, and inflammatory indicators such as CRP, IL-6, TNF-alpha, IgA, and cortisol. Among the 12 included trials, 8 referred to serum CK level. Time to peak of serum CK ranged from 24 h to 7 d. The majority of included studies [59, 61, 62, 66, 68] indicated a nonsignificant difference of circulating CK between *n*‐3 PUFA and control groups while others revealed a downregulated CK level [58, 63, 64] or a delayed time to peak in the *n*‐3 PUFA group [64].

CRP level was assessed in 3 studies. Corder et al. [57] evaluated CRP concentration of saliva while DiLorenzo et al. [58] measured CRP level in serum, both of which demonstrated no significance between the *n*‐3 PUFA and control groups, although Phillips et al. [66] observed that the treatment group had a reduction in serum CRP of borderline significance compared to the placebo group.

Serum IL-6 was analyzed by 7 studies. Both Phillips et al. [66] and Tsuchiya et al. [68] reported that serum IL-6 reached the peak at 3 d postexercise. Four out of 7 studies found a significant decrement in the *n*‐3 PUFA groups compared to the placebo groups [58, 66–68], while others exhibited no differences [61, 62] or even an upregulated IL-6 level [60].

Data on TNF-alpha level was presented in 3 studies, 2 of which indicated no effect of *n*‐3 PUFA on blood TNF-alpha level [62, 68], while 1 exhibited a significantly lower level in an experimental group after 2 h postexercise [64].

Two studies tested the cortisol level with conflicting results. One study reported a significant reduction of cortisol level in the *n*‐3 PUFA group at both 24 hours and 48 hours after exercise [63], while the difference was not observed in the other study [62].

*n*‐3 PUFA was reported to have no significant effect on other blood indicators, including IL-1ra [58], iron [62], and LDH [66]. On the other hand, IgA concentration was reported to be significantly lower in the *n*‐3 PUFA group, and the IgA peak time was also 24 h earlier in the *n*‐3 PUFA group [63]. In addition, blood troponin I and myoglobin peaked at 24 h and 72 h, respectively, after eccentric exercise, and both of them attenuated significantly in the *n*‐3 PUFA groups [64].

#### 3.5.2. Muscle Swelling

Seven included studies have stated the effect of *n*‐3 PUFA on postexercise muscle swelling which was assessed by measuring upper arm circumference or thigh circumference. Arm circumference increased by ~2% after eccentric exercise, suggesting that swelling/edema was present [60]. However, except for Lenn et al. [62] who reported PUFA's preventive role in increased upper arm circumference after exercise and Tartibian et al. [67] who exhibited the opposite trend in thigh circumference, most articles illustrated no significant effect of *n*‐3 PUFA in upper arm/thigh circumferences [57, 63–65, 68].

#### 3.5.3. Skin Temperature

For skin temperature evaluation, only one study conducted by Corder et al. [57] presented no significant change between baseline and 48 h follow-up in either group, which indicated that inflammatory response in local muscle did not increase skin temperature.

#### 3.5.4. Jump Performance

With regard to jump performance, Jakeman et al. [61] found that participants who consumed a large dose of *n*‐3 PUFA had better squat jump performance as well as countermovement jump performance than the control subjects.

#### 3.5.5. M-Wave Latency

Only Ochi et al. measured M-wave latency of the biceps brachii to evaluate motor nerve function [65]. The outcome indicated that M-wave latency was significantly longer in the control group than in the*n*‐3 PUFA group immediately after the exercise.

#### 3.5.6. GRADE approach

The evidence profile of different outcome measurements using the GRADE approach is shown in Table 2. Since only RCTs were considered for this meta-analysis, an initial rating of “high” was assigned to all outcome parameters which could then be downgraded according to the presence and seriousness of the following five factors: risk of bias, inconsistency, imprecision, indirectness, and publication bias. In our current meta-analysis, the certainty of evidence for all the three outcome parameters was downgraded by two levels due to critical study limitation (the majority of studies had study design features that carried a high risk of bias due to attrition bias) and imprecision (the total number of participants for each outcome parameter was less than 400).

## 4. Discussion

The current systematic review and meta-analysis with 12 RCTs involving 145 experimental subjects and 156 controls suggested that *n*‐3 PUFA supplementation was associated with less muscle soreness after exercise, but *n*‐3 PUFA supplementation did not influence isometric muscle strength as well as ROM. Furthermore, since no consensus has been reached on the optimal dosage of *n*‐3 PUFA and supplement duration, subgroup analyses based on dosage and supplement duration were also undertaken, with corresponding outcomes indicating that dosage as with supplement duration did not influence the effects significantly. However, we found that the pooled effect size of pain reduction was lower than the MCID of 1.4 units that we used in our current meta-analysis. Thus, the statistically significant difference of DOMS favoring *n*‐3 PUFA supplementation was very unlikely to equate to meaningful or important changes in practice. Besides, we also summarized the actions of *n*‐3 PUFA on some other secondary outcomes like blood indicators and upper arm/thigh circumferences. But because of insufficient data as well as heterogeneity of treatments and endpoint measures, these secondary outcomes were not appropriate for quantitative data syntheses.

For decades, researches have demonstrated the interdependent association between physical fitness and cardiovascular disease risk status among adults [70, 71], which, of note, has also been reported in children and adolescents [72], persisting even after adjustment for fatness as well as other confounding factors [73] and could track moderately well from childhood and adolescence into adulthood [74]. Considering the indisputable beneficial effects of physical exercises, the American College of Sports Medicine (ACSM) recommends a comprehensive program of exercise including cardiorespiratory, resistance, flexibility, and neuromotor exercise of sufficient volume and quality for apparently healthy adults of all ages [75]. As a matter of fact, it might be difficult at the beginning for those who remain exercise inactive, and DOMS is among the most common excuses for not participating. Under such circumferences, to alleviate such discomfort with dietary constituents and supplements would be a far more appealing choice, since for most healthy individuals, professional physical therapy and NSAID medication might be more like a fuss.

*n*‐3 PUFA, well known for its anti-inflammatory effect, has been examined extensively for a great range of health outcomes as well as prevention of a wide array of diseases. As a nutritional factor that may reduce pain caused by various incentives, *n*‐3 PUFA also received lots of attentions for the treatment of exercise-related muscle damage. This study provided combined evidence to show that temporal intake of isolated *n*‐3 PUFA could alleviate DOMS caused by strenuous exercises regardless of differences in the eccentric exercise protocols and/or muscle groups used, although no statistical difference was observed in ROM and isometric muscle strength. Moreover, effects on MVC and upper arm/thigh circumferences still remained equivocal, which might be due to the aforementioned differences in supplementation dose/duration as well as varying modes of exercise. In this respect, though there was insufficient evidence to say that *n*‐3 PUFA could alter the condition of edema/swelling and muscle damage, or physical performances postexercise, its protective role in alleviating DOMS after eccentric exercise was somehow obvious.

Both *n*‐3 PUFA and *n*‐6 PUFA belongs to PUFA family, which have two fundamental physiological functions in humans. On the one hand, they present as structural phospholipids in all cytomembranes and contribute to membrane functions. On the other hand, they are the primary precursors of the eicosanoids. However, *n*‐3 PUFA and *n*‐6 PUFA are transformed into corresponding eicosanoids each with exclusive functions. *n*‐3 PUFA is believed to decrease the activity of *n*‐6 PUFA arachidonic acid (ARA), a fatty acid that is related to the production of inflammatory mediators [76]. Though we did not perform meta-analysis of blood cytokine levels due to divergent evaluating methods, *n*‐3 PUFA appeared to have limited effects from the summarized results mentioned above, and whether this results from a considerably short supplement duration of*n*‐3PUFA or suggests different mechanisms involved in the release of cytokines and muscle injury still remain to be further discussed.

A previous meta-analysis showed that *n*‐3 PUFA could blunt inflammatory joint pain, though to gain a therapeutic effect, a minimum of 3 months' supplement duration at a dose of 2.7 g*n*‐3 PUFA was required [77]. Still, another meta-analysis exhibited a moderate analgesic effect of *n*‐3 PUFA in chronic pain, especially dysmenorrhea. Furthermore, a larger effect was observed for low doses and short periods of treatment [78]. When it comes to healthy individuals, though without evidences, it has been far long recommended by clinicians for different populations including athletes to consume 1000-2000 mg per day of *n*‐3 PUFAs [79]. As majority of previous studies utilized daily large doses of *n*‐3 PUFA (>1000 mg) to reveal their biological functions in inflammatory diseases and cancer, as well as in human health in general [80], this study went further by discussing the influences of both large (>1000 mg/d) and small (<1000 mg/d) dosage supplements, as well as >1 w and <1 w intake duration, and came out with the idea that the effect of *n*‐3 PUFA on DOMS might not be dose- and duration-dependent, indicating a possibly more cost-effective way to ameliorate DOMS.

In order to assess the quality of evidence from our included studies, we used the GRADE approach which takes five categories of limitations (risk of bias, indirectness, inconsistency, imprecision, and publication bias) into consideration. All the included RCTs were initially rated as “high quality” and then downgraded based on the presence and seriousness of the limitations. We downgraded the quality of evidence for all outcome measurements by one level due to the high risk of bias (the majority of studies did not report adverse events associated with *n*‐3 PUFA supplementation). No downgrading was performed for indirectness. No downgrading was done for all the three outcomes since the estimate of heterogeneity was acceptable (DOMS: *I*^2^ = 39%; isometric muscle strength: *I*^2^ = 3%; and ROM: *I*^2^ = 16%). Downgrading for imprecision was supported by a relatively limited number of participants, since none of the outcomes had participants of more than 400. Begg's test along with Egger's test suggested no publication bias. In summary, all the three outcomes were graded as low quality. In other words, further research is very likely to have an important impact on our confidence in the estimate of effect and is likely to change the estimate. Considering the clinically marginal effect of *n*‐3 PUFA supplementation on DOMS, despite the low-quality evidence from the included RCTs, it is still too early to recommend to young sport beginners to take *n*‐3 PUFA supplements to reduce muscle soreness after exercise.

Although we have collected existing evidences based on RCTs and provided a more comprehensive evaluation, there are still several aspects waiting to be improved in the current study. Firstly, the studies included contained relatively small sample sizes, resulting in reduced statistical power. Secondly, based upon the findings of our current meta-analysis, the effect of *n*‐3 PUFA supplementation on muscle soreness appeared to be clinically marginal. Furthermore, we failed to further achieve a better choice of supplement dosage or duration due to the limited number of included studies. Also, risk of bias assessment indicated that the majority of studies had flaws in study design and implementation (most studies did not report adverse events associated with *n*‐3 PUFA supplementation). Thirdly, with the exception of Lenn et al. [62], all included trials studied only male or female subjects, thus reducing the generalization of the outcome. Lastly, subjects were basically young adults who practically participated in no resistance training within 6 months, so the effect in athletes or in the middle aged and elderly remain to be further discussed. Under such scenario, we call for large-scale, well-modified RCTs which carefully evaluate dose and duration-response relationships and involve roles of *n*‐3 PUFA on DOMS as well as other indicators of muscle damage and inflammatory status.

## 5. Conclusion

Low quality of evidence suggested that *n*‐3 PUFA supplementation was associated with significantly reduced DOMS after eccentric exercise, but the reduction in pain was unlikely to be clinically important. There is low-quality evidence that *n*‐3 PUFA does not improve isometric muscle soreness and range of motion after exercise. Existing evidence on the potential preventive effect of PUFA on other measurements of muscle damage and inflammatory status are too limited to lead to conclusions. Taken together, taking *n*‐3 PUFA supplements is not an effective way to reduce DOMS after eccentric exercise.

## Figures and Tables

**Table 1:** Main characteristics of included RCTs.

Study	Country	Mean age	Gender	Cohorts	Intervention and control	Eccentric exercise	Main outcomes	AEs
Corder et al., 2016	USA	31.9/33.3	F	14/13	O: 3000 mg DHA daily, for 7 d before exercise and 2 d afterwards C: placebo capsules (corn and soy oil)	T: 4 sets preacher bench biceps curl W: 120% of subjects' 1 RM D: over 4 s in a controlled manner R: till failure	DOMS, ROM, upper arm circumference, skin temperature, and blood markers	None

DiLorenzo et al., 2014	Virginia	21.8/21.8	M	21/20	O: 2000 mg DHA daily, for 28 d before exercise C: placebo capsules	T: 6 sets preacher bench curl W: 140% of subjects' 1 RM D: with the investigators 1-5 counting R: 10 reps/set	DOMS, ROM, muscle strength, and blood markers	None

Gray et al., 2014	UK	22.0/23.0	M	10/10	O: 1300 mg EPA and 300 mg DHA daily, for 6 w before exercise C: placebo capsules (olive oil)	T: 20 sets knee extension W: NR D: 0.52 rads/s between 90° and 120° R: 10 reps/set	DOMS, MVC, and blood markers	NR

Houghton and Onambele, 2012	UK	20.4/20.4	F	7/10	O: 360 mg EPA daily, for 3 w before exercise and 6 w afterwards C: placebo capsules (lecithin)	T: 4 sets leg flexion and extension walking lunges straight leg deadlift W: 70% of subjects' 1 RM D: within 6 s R: 10 reps/set	DOMS, muscle strength, and blood markers	NR

Jakeman et al., 2017	UK	25.6/26.2	M	9/9	O: 750 mg EPA and 50 mg DHA, immediately after the exercise C: placebo capsules (filler oil)	T: 10 sets plyometric drop jump W: body weight D: 10 s between each jump R: 10 reps/set	DOMS, muscle strength, jump performance, and blood markers	NR

Lenn et al., 2002	USA	23.4/23.4	M/F	7/7	O: 1800 mg Omega-3 fatty acid daily, for 30 d before exercise and 7 d afterwards C: placebo capsules (Western fat blend)	T: arm training W: subjects' 1 RM D: 1 s at 90°/s R: 50 reps in total	DOMS, MVC, ROM, RANG, upper arm circumference, and blood markers	NR

Meamarbashi and Abedini, 2011	Iran	18.2/18.2	M	10/10	O: 1200 mg purslane extract daily, for 3 d before exercise and 2 d afterwards C: placebo capsules (lactose powder)	T: 7 sets bench stepping W: body weight D: 4 s R: 75 reps/set	DOMS, ROM, muscle strength, thigh circumference, and blood markers	NR

Mickleborough et al., 2015	USA	21.7/21.5	M	16/16	O: 58 mg EPA and 44 mg DHA and 0.225 mg vitamin E daily, for 26 d before exercise and 4 d afterwards C: placebo capsules (olive oil)	T: downhill running W: at -16% grade D: in total 20 min R: NR	DOMS, MVC, ROM, PPT, and blood markers	None

Ochi et al., 2017	Japan	20.7/21.3	M	10/11	O: 600 mg EPA and 260 mg DHA daily, for 8 w before exercise and 5 d afterwards C: placebo capsules (corn oil)	T: 6 sets preacher bench curl W: 40% of subjects' 1 RM D: 3 s at a constant velocity of 30°/s R: 10 reps/set	DOMS, MVC, ROM, upper arm circumference, and M-wave latency	NR

Phillips et al., 2003	USA	22.1/22.1	M	20/20	O: 800 mg DHA and 300 mg tocopherols and 300 mg flavonoids daily, 7 d before exercise and 7 d afterwards C: placebo capsules (sunflower oil and rice powder)	T: 3 sets arm curl W: 80% of subjects' 1 RM D: over 6 s R: 10 reps/set	DOMS, ROM, and blood markers	None

Tartibian et al., 2009	Germany	33.4/33.5	M	9/18	O: 324 mg EPA and 216 mg DHA daily, for 30 d before exercise and 2 d afterwards C: placebo capsules	T: 7 sets bench stepping W: body weight D: 4 s R: 75 reps/set	DOMS, ROM, and thigh circumference	NR

Tsuchiya et al., 2016	Japan	19.4/19.5	M	12/12	O: 600 mg EPA and 260 mg DHA daily, for 8 w before exercise and 5 d afterwards C: placebo capsules (corn oil)	T: 5 sets elbow flexion W: 6 RM D: 3 s at a constant velocity of 30°/s R: 6 reps/set	DOMS, MVC, ROM, upper arm circumference, and blood markers	NR

Cohort size and mean age of subjects were presented as Omega-3 group/control group. M: male; F: female; O: Omega-3 group; C: control group; DHA: docosahexaenoic acids; EPA: eicosapentaenoic acid; RM: repetition maximal; T: type of eccentric exercise; W: weight of eccentric contraction; D: duration of each eccentric contraction; R: repetitions for each set of eccentric exercise; DOMS: delayed onset muscle soreness; MVC: maximal voluntary contraction; ROM: range of motion; AEs: adverse events; NR: not reported.

**Table 2:** Summary of findings from RCTs on the use of *n*‐3 PUFAs for the treatment of muscle soreness after eccentric exercise.

Outcome	No of studies (participants)	ROB	Indirectness	Inconsistency	Imprecision	Publication bias	Pooled effect	*I* ^2^	GRADE
DOMS	9 (210)	Serious^a^	Not serious	Not serious	Serious^b^	Not serious	MD: -0.93 (-1.44, -0.42)	39%	Low
Isometric muscle strength	6 (144)	Serious^a^	Not serious	Not serious	Serious^b^	Not serious	SMD: -0.03 (-0.36, 0.31)	3%	Low
ROM	4 (93)	Serious^a^	Not serious	Not serious	Serious^b^	Undetected	SMD: -0.21 (-0.24, 0.67)	16%	Low

Moderate quality: further research is likely to have an important impact on our confidence in the estimate of effect and may change the estimate. Low quality: further research is very likely to have an important impact on our confidence in the estimate of effect and is likely to change the estimate. Very low quality: we are very uncertain about the estimate. RCT: randomized controlled trial; ROB: risk of bias; DOMS: delayed onset muscle soreness; ROM: range of motion; MD: mean difference; SMD: standard mean difference; *n*‐3 PUFA: Omega-3 polyunsaturated fatty acid. ^a^Downgraded as most of the included RCTs had critical limitation for attrition bias. ^b^Downgraded for continuous variables if the sample size was less than 400.

## Data Availability

This is a systematic review and meta-analysis of published papers, and data could be found online.

## Abbreviations

*n*‐3 PUFA: Omega-3 polyunsaturated fatty acid
EPA: Eicosapentaenoic acid
DHA: Docosahexaenoic acid
DOMS: Delayed onset muscle soreness
ROM: Range of motion
RCT: Randomized controlled trial
SMD: Standard mean difference
MD: Mean difference
95% CI: 95% confidence interval.
